# Developing an Improved Cycle Architecture for AI-Based Generation of New Structures Aimed at Drug Discovery

**DOI:** 10.3390/molecules29071499

**Published:** 2024-03-27

**Authors:** Chun Zhang, Liangxu Xie, Xiaohua Lu, Rongzhi Mao, Lei Xu, Xiaojun Xu

**Affiliations:** Institute of Bioinformatics and Medical Engineering, School of Electrical and Information Engineering, Jiangsu University of Technology, Changzhou 213001, China; zhangchlwy@gmail.com (C.Z.); xieliangxu@jsut.edu.cn (L.X.); jsluxiaohua@outlook.com (X.L.); maorongzhi2022@outlook.com (R.M.)

**Keywords:** deep learning, molecule generation, bidirectional, CycleGAN, attention

## Abstract

Drug discovery involves a crucial step of optimizing molecules with the desired structural groups. In the domain of computer-aided drug discovery, deep learning has emerged as a prominent technique in molecular modeling. Deep generative models, based on deep learning, play a crucial role in generating novel molecules when optimizing molecules. However, many existing molecular generative models have limitations as they solely process input information in a forward way. To overcome this limitation, we propose an improved generative model called BD-CycleGAN, which incorporates BiLSTM (bidirectional long short-term memory) and Mol-CycleGAN (molecular cycle generative adversarial network) to preserve the information of molecular input. To evaluate the proposed model, we assess its performance by analyzing the structural distribution and evaluation matrices of generated molecules in the process of structural transformation. The results demonstrate that the BD-CycleGAN model achieves a higher success rate and exhibits increased diversity in molecular generation. Furthermore, we demonstrate its application in molecular docking, where it successfully increases the docking score for the generated molecules. The proposed BD-CycleGAN architecture harnesses the power of deep learning to facilitate the generation of molecules with desired structural features, thus offering promising advancements in the field of drug discovery processes.

## 1. Introduction

The cornerstone of novel drug design resides in the exploration of small molecules that meet defined criteria, a task that has historically presented challenges [1,2,3]. Traditionally, this endeavor has heavily depended on the expertise of professionals involved with the processes of screening, synthesis, and testing, incurring considerable demands on both human resources and materials. Computer-aided drug design (CADD) has brought about improvements to this situation [4,5,6,7,8,9,10,11], culminating in notable achievements thus far. Especially since the emergence of artificial intelligence, deep learning-based molecule generation has brought about achievements as one of the advanced molecular modeling techniques. Recently, deep learning-designed drugs have entered pre-clinical trials [12,13,14,15,16,17].

In the field of computer-aided drug design (CADD), machine learning and deep learning have gained significant attention. Machine learning involves the development of algorithms that enable computers to learn and make predictions without explicit programming. Deep learning, a subset of machine learning, employs artificial neural networks with multiple layers to extract complex patterns from data. One important application of deep learning in CADD is the use of deep generative models. Deep generative models are designed to generate new data samples that resemble a given training dataset. In the context of drug discovery, these models play a crucial role in de novo molecule generation. De novo molecule generation refers to the process of designing and synthesizing entirely new molecules with desired properties.

Deep learning shows the power of prediction and generation. To make use of its generation ability, de novo molecular design methods have been developed [18,19]. These methods are based on recurrent neural networks, variational autoencoders (VAEs), generative adversarial networks (GANs), and transformers, etc. For example, Pham et al. employed conditional variational autoencoder frameworks to efficiently generate novel molecules with enhanced biological activity [20]. Wang et al. utilized generative pre-training techniques to extract contextual information from molecules, facilitating the generation of molecules with improved binding affinity to target proteins [21].

**Figure 1:**
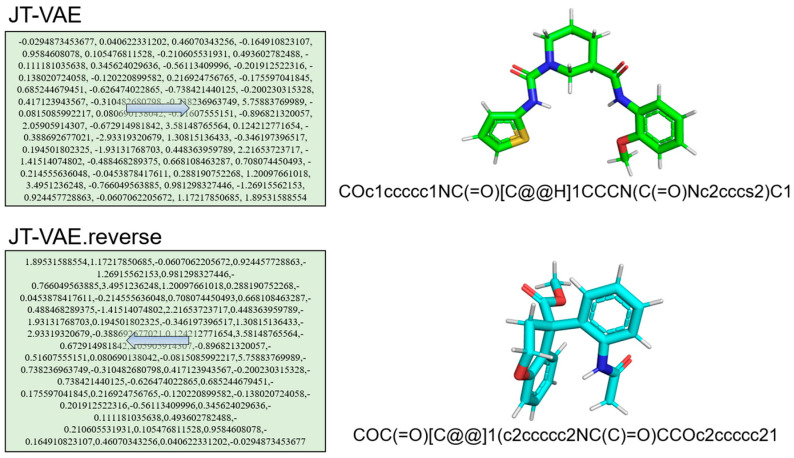
The schematic figures of processing of forward and reverse of JT-VAE. Reading the input from two directions (labelled as arrows) would generate different SMILES strings. The left panel shows the reading of the JT-VAE encoding in a forward and backward direction; and the right panel shows the corresponding molecules.

Among these widely reported methods, generative adversarial network (GAN) has emerged as a popular architecture for generating highly realistic molecules [22,23]. By simulating the potential distribution of molecules through the interplay between generators and discriminators, GAN can generate a diverse range of molecular structures. In the field of de novo molecular generation, GAN exhibits distinctive characteristics with an ability to construct variants by changing component architecture in generators and discriminators. Consequently, a multitude of GAN architectures are being explored and developed to cater to diverse requirements. The combination of reinforcement learning and GANs enables control over the attributes of generated samples, facilitating the generation of sequence data that aligns with specific target requirements [24]. LatentGAN directly incorporates the autoencoder into the GAN, employing the pre-trained autoencoder to map the molecular structure to potential vectors and to train the GAN using the potential vectors as inputs and outputs [25]. Beyond from GAN, CycleGAN employs a ring network comprising two sets of symmetric generators and discriminators, enabling dual unpaired data transformations during the generation process [26]. The Mol-CycleGAN [27] model extends CycleGAN to the junction tree variational autoencoder (JT-VAE) [28] framework to ensure that the generated compounds are always effective. The original molecular data set is fed into the “codec” structure to generate new compounds with desired pharmacological properties [27,28,29]. Similar to the natural language, the forward and backward directions of the input molecule would preserve sequential information as shown in Figure 1. Therefore, information from reading the input molecules from the forward direction and the backward direction should be kept.

**Figure 2:**
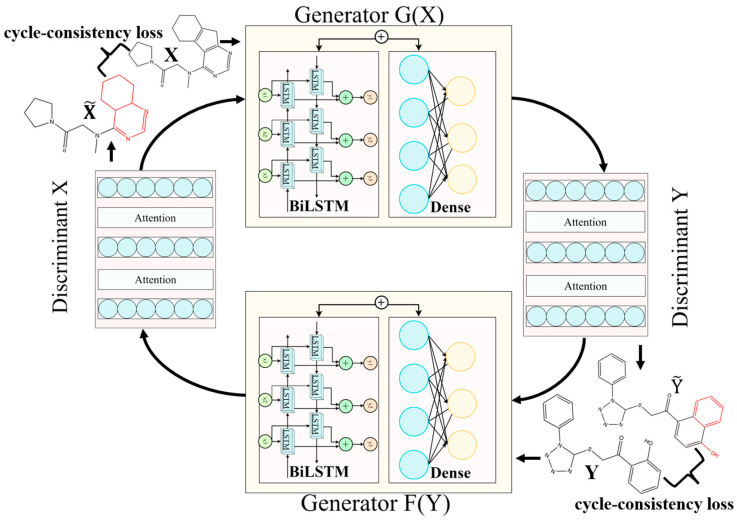
The proposed architecture of the BD-CycleGAN. Discriminators consist of three dense layers that connected by two Attention layers. Generators consist of one BiLSTM layer, one Dense layer and the residual connection between two layers. The cycle-consistency loss and molecules are displayed on both sides of the schematic figure to demonstrate the loss in models.

Herein, we propose BD-CycleGAN (Figure 2), which incorporates bi-directional long short-term memory (BiLSTM) [30] and attention mechanisms [31] into the Mol-CycleGAN generator and discriminator, respectively. BiLSTM is a type of recurrent neural network that can process information in both the forward and backward directions. The CycleGAN model is a type of deep learning model that learns to transform molecules from one form to another. In our case, it learns to generate new molecules with the desired structural features. The attention mechanism is a component used in deep learning models. It allows the model to focus on different parts of the input data while performing a task, giving more weight or attention to relevant information. Our proposed BD-CycleGAN architecture combines the strengths of the CycleGAN model, BiLSTM, and an attention mechanism to enable the generation of new molecules in drug discovery. The inclusion of BiLSTM within the generator allows for the capture of sequential context information, enabling better modeling of molecular sequence features and enhancing the generator’s capability to express molecular structures. The generated molecules exhibit higher potential for the lead candidates and align more closely with the principles of pharmaceutical chemistry. The improvement in the performance of the optimized model is evaluated by the success rate, diversity, and novelty indicators.

## 2. Results

### 2.1. Ablation Experiment to Identify Architecture of Model

**Figure 3:**
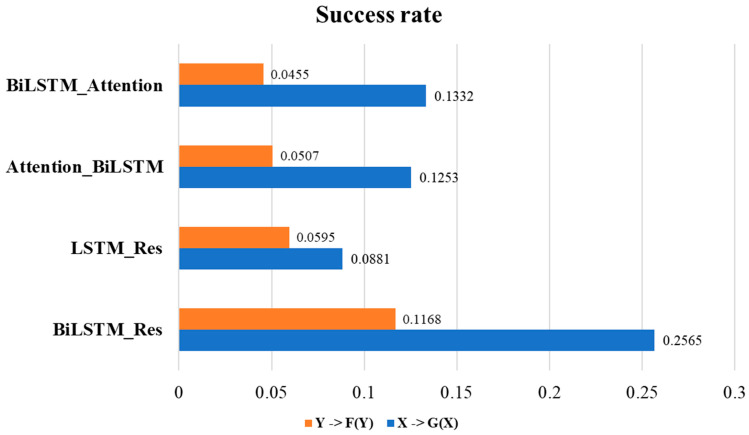
Success rate results for the four combined models in the ablation experiment.

We conduct ablation experiment on our proposed model by using the generation task of aromatic data set. To enhance the generative ability, we need to revise the generator architecture of CycleGAN. The architecture of four combination models, including BiSTM_Attention, Attention_BiLSTM, LSTM_Res, and BiLSTM_Res, are presented in Figure S1 in the Supplementary Materials Section. Figure 3 presents the success rate of the four examined models. The success rate of BiLSTM_Res is higher than that of the other three models, suggesting that the molecule generation ability of BiLSTM_Res model surpasses that of the other modules. Figure S2 shows that the BiLSTM_Res model generates a higher number of target characteristic molecules compared to the other modules combinations.

In our ablation experiment, we firstly show that one layer of BiLSTM could outperform two layers of LSTM as shown in Figure 3. Secondly, the attention mechanism and residual connection are embedded in the discriminator to assist in focusing on key features. Comparing BiLSTM_Attention and BiLSTM_Res, we can see that the BiLSTM_Res will build the connection between the output of the BiLSTM and the input, which can provide more information after dimensionality reduction and will improve gradient transfer and enhance the model’s learning capacity. Thereby, BiLSTM_Res enhances its discriminative ability and guides the generator in generating more realistic and diverse molecules. As a result, BiLSTM_Res is selected as the generator module for our proposed BD-CycleGAN models.

### 2.2. Molecular Generation with Specific Structural Group

**Figure 4:**
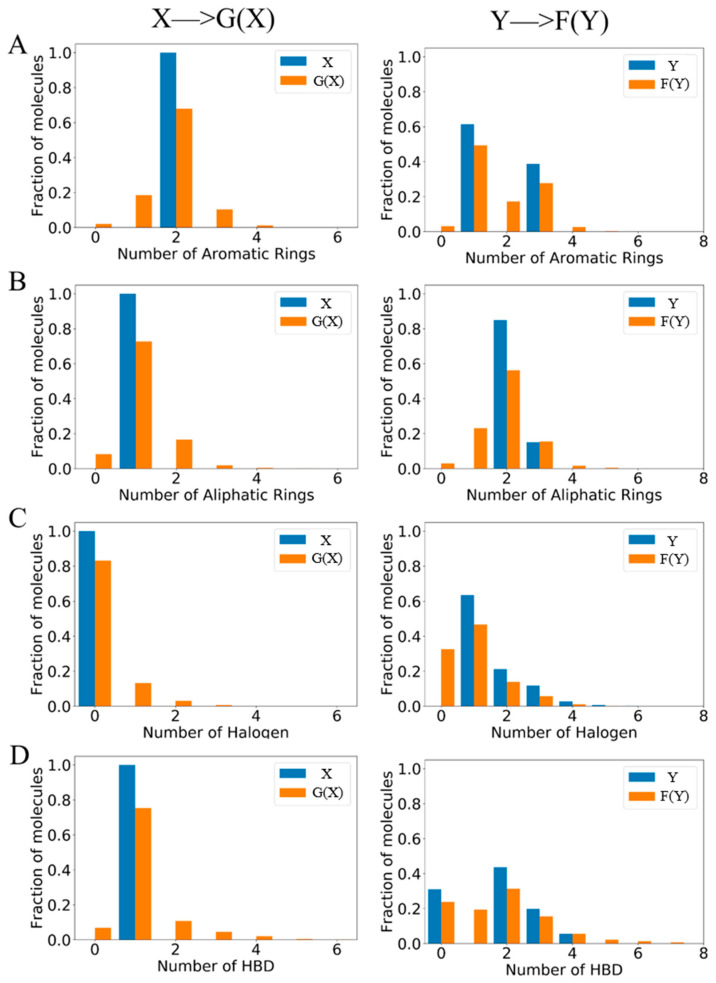
The distribution of the generated molecules. (**A**) Aromatic rings, (**B**) aliphatic rings, (**C**) halogen, and (**D**) HBD. In each sub-figure, the blue bars represent the original molecular distribution and the orange bars represent the generated molecular distribution.

**Figure 5:**
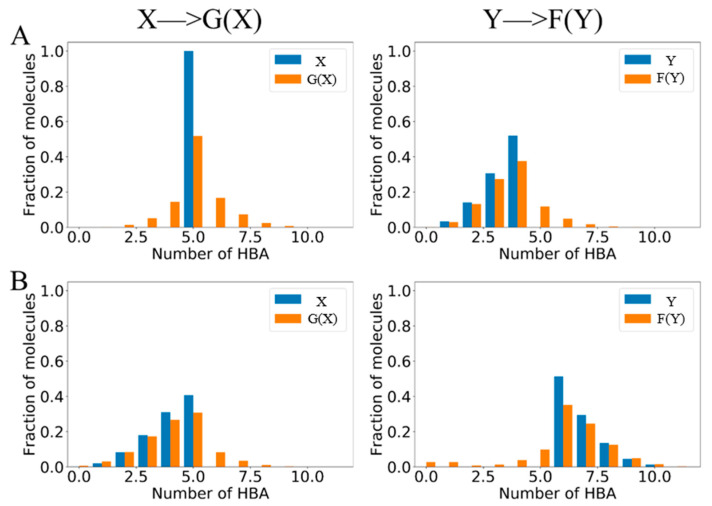
The distribution of the generated molecules for hydrogen bond acceptors. (**A**) HBA_Discrete and (**B**) HBA_Continuous. “HBA_Discrete” refers to the discrete representation of the number of hydrogen bond acceptors. “HBA_Continuous” refers to the continuous numerical values of the number of hydrogen bond acceptors. In each sub-figure, the blue bars represent the original molecular distribution and the orange bars represent the generated molecular distribution.

In the lead optimization process, the pharmacodynamic functional groups need to be tuned to change the property of potential molecules. BD-CycleGAN is proposed to complete this task to transform from source molecules to target molecules. CycleGAN adopts the special cycle architecture of complete symmetry. The model will transform and reconstruct the molecular data in the two relative regions of X and Y, to realize the transformation and generation of molecular structure. As shown in Figure S3, the ZINC-250K data set was divided into six data sets based on the number of five functional groups: aromatic ring, aliphatic ring, halogen, hydrogen bond donor (HBD), and hydrogen bond acceptor (HBA). Figure 4 shows the molecular distribution of the different structural features of the generated aromatic ring, aliphatic ring, halogen, and hydrogen bond donor (HBD). Compared with molecule set X, which is the original molecular distribution, it is obvious that more generated molecules are close to the characteristics of molecule set Y. The molecules have been successfully converted from the source molecule X to the target molecule Y. The transformation is more obvious in the task of HBA_Discrete generation (shown in Figure 5). The X set only contains molecules with five hydrogen bond acceptors, while the Y set contains molecules with fewer than five hydrogen bond acceptors. The results show that the success rate of converting a single feature into multiple continuous features is improved.

We employed two key evaluation criteria, the ability of the model to generate the required molecules and the likelihood of successfully generating molecules with the desired functional groups. We have presented the corresponding results in Figure 4 and Figure 5, and summarized them in Table 1. The results demonstrate our model can generate the desired molecules.

### 2.3. Performance Evaluation on the Chemical Structure

The structural performance of the model is quantitatively calculated under six different feature distributions: aromatic ring, aliphatic ring, halogen, HBD, HBA_Discrete, and HBA_Continuous. The generator and discriminator are divided into two directions, so the generation of two directions is realized. The evaluation indicators for the two directions (X—>G(X) and Y—>F(Y)) are summarized in Table 1. The BD-CycleGAN model has been improved in terms of success rate, uniqueness, and non-identity. The success rate and non-identity of the aromatic ring are improved. The diversity has been improved for aromatic ring, HBD, and HBA_Discrete. Since the aromatic ring structure has a long chain-like structure, it is necessary to deal with and transfer the long-term dependence and complex spatial structure during the simulation of molecular generation. BiLSTM and the attention mechanism can deal with this situation well, to improve the processing ability and accuracy of the molecular generation model for the aromatic ring structure.

For the halogen feature distribution, the success rate obviously improved from 0.032 to 0.121 for G(X) and from 0.145 to 0.257 for F(Y). The non-uniformity is nearly doubled, indicating that the model has a good advantage in dealing with structures with halogens. For the HBA_Discrete distribution, the molecules in the X set only have samples with five hydrogen bond acceptors. This specific restriction allows the model to capture the contextual information of the input molecules more accurately and to generate molecules that are better matched with the characteristics of the target. Therefore, the BD-CycleGAN model achieves a doubling of the success rate and non-identity in the generation task of the HBA_Discrete distribution.

To quantitatively assess the performance of the proposed model, the evaluation matrices are compared between BD-CyleGAN and Mol-CycleGAN using the MOSES benchmark. As shown in Table 2, the model was analyzed using five indicators: Filters, Valid, IntDiv, IntDiv2, and Novelty to determine the effectiveness of the model’s molecular generation performance, and the diversity of the generated molecules. Among the six different feature distributions, the halogen task has the highest novelty, which means that the resulting molecules have more unique structural properties. Meanwhile, the aliphatic rings generation scored the highest, indicating that the resulting molecules are more stable in structural generation, which may have a wider range of applications and greater research value. In the filter evaluation, HBD had the highest score and the best performance. In the evaluation of molecular effectiveness, the six different distributions were significantly improved, which means that the BD-CycleGAN model can better learn the distribution of functional groups during the training process so that the generated molecules are more in line with the requirements of functional group distribution. Such molecules are more in line with the law of medicinal chemistry and biological feasibility and may be easier to synthesize and apply in practical treatment.

### 2.4. Structure and Property Analysis

To evaluate the quality of the molecular generation models, one of the most important tasks is to evaluate the similarity of molecular generation. Selecting the appropriate similarity index is of great significance for evaluating the quality of the model and optimizing the generated molecules. In this paper, the similarity between the molecules generated by the BD-CycleGAN model and the molecules of the original dataset was evaluated by visualizing the Tanimoto similarity, visualizing the molecules, and the four indicators FCD, SNN, Scaff, and Frag in the MOSES platform.

**Figure 6:**
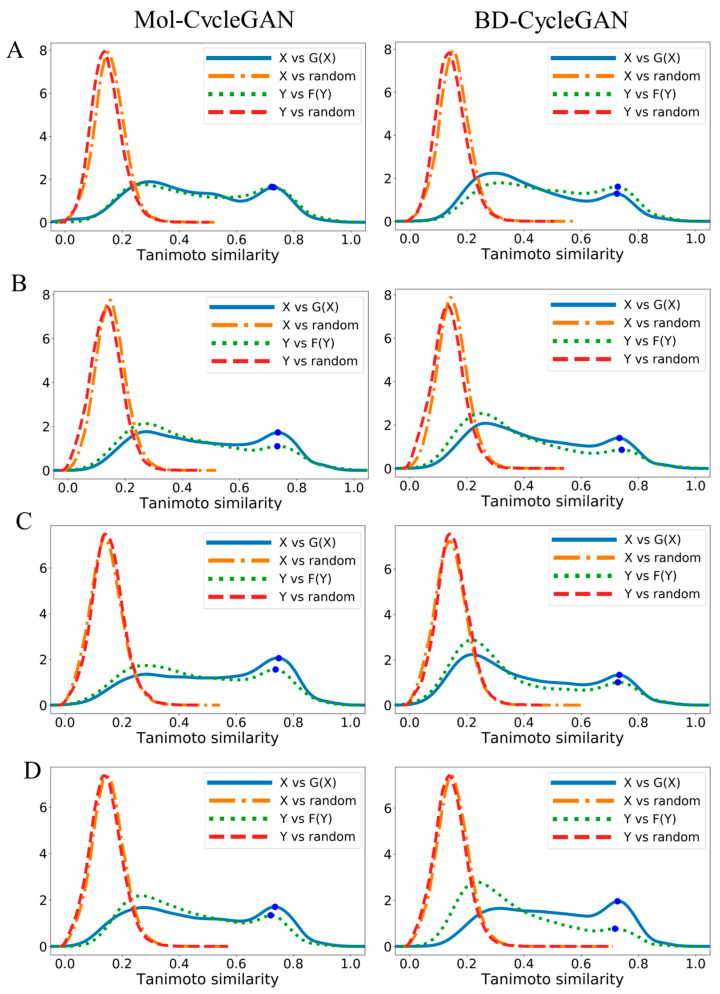
Density map of Tanimoto similarity. (**A**) Aromatic rings, (**B**) aliphatic rings, (**C**) halogen, and (**D**) HBD. X and Y are the original molecules and G(X) and F(Y) are the generated molecules. “random” refers to the molecules that were randomly selected from the ZINC-250K dataset. Blue dots represent the location of distribution peaks.

**Figure 7:**
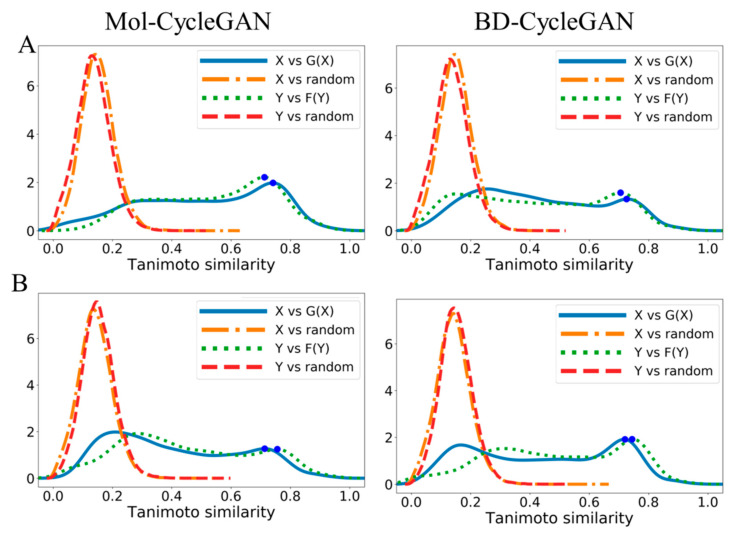
Density map of Tanimoto similarity. (**A**) HBA_Discrete and (**B**) HBA_Continuous. X and Y are the original molecules and G(X) and F(Y) are the generated molecules. “random” refers to the molecules that were randomly selected from the ZINC-250K dataset. Blue dots represent the location of distribution peaks.

As shown in Figure 6 and Figure 7, we performed Tanimoto similarity analysis on datasets containing different functional group distributions. We can intuitively see that the BD-CycleGAN model has slightly lower similarity in the distribution of aromatic rings, aliphatic rings, and halogen than the Mol-CycleGAN model. HBD and HBA_Continuous distributions show an improved similarity. The embedded BiLSTM and attention make the model pay more attention to local structures and specific features while pursuing generative diversity and novelty. Meanwhile, some features with similar overall structures may be ignored. This makes the overall similarity of molecules generated by the BD-CycleGAN model slightly reduced. However, the HBA and HBD features in a molecule are often related to the local structure of the molecule, so the BD-CycleGAN model can generate HBA and HBD features that are more accurate and closer to the original molecule, thereby improving their similarity. Such improvements have implications for studying molecular similarity and drug design from a biological perspective.

Table 3 and Table 4 show the four evaluation metrics, FCD, SNN, Frag, and Scaff, used to evaluate the similarity of the generated molecules. The test set is divided from the ZINC-250K dataset and TestSF is from the test_scaffold of MOSES. As shown in Table 3, the generated molecules with hydrogen bonds performed significantly better. Because the formation of hydrogen bonds (both HBA and HBD) usually involves specific interactions between atoms, BD-CycleGAN is better at modeling the sequences, so the similarity between HBA and HBD is higher. The similarity between aromatic and aliphatic rings, on the other hand, may be more dependent on the structure of the ring and the type of bond, and thus the similarity is relatively poor.

**Figure 8:**
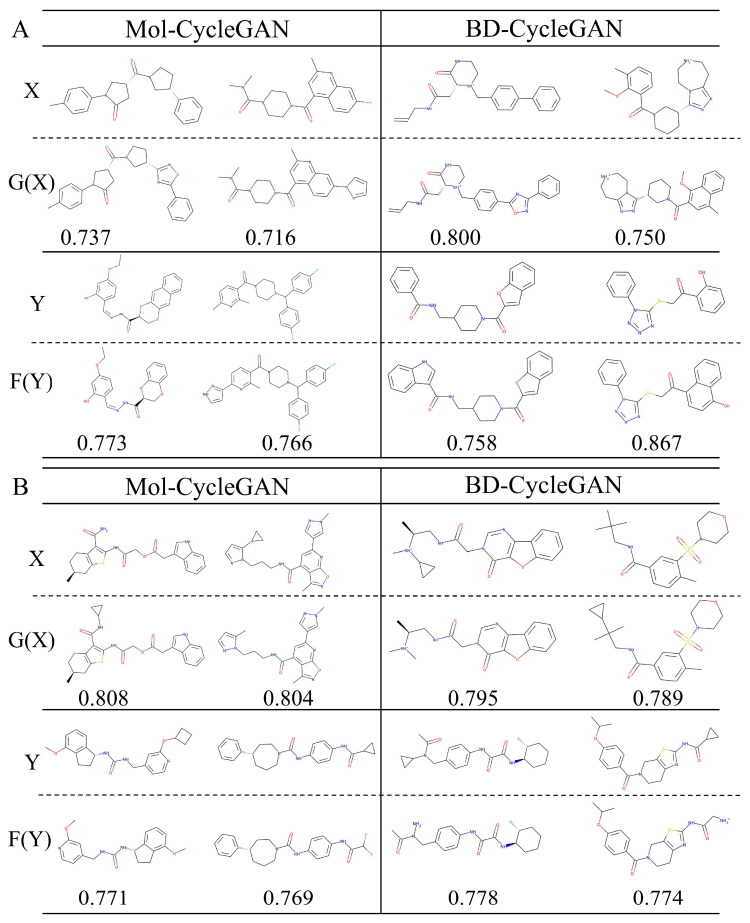
Structure diagram of the original and generated molecules. The molecules are generated in a cyclic manner. The model generates molecules G(X) based on input X and obtains the pharmacophore property of Y, and vice versa. X and Y are the original molecules. G(X) and F(Y) are the generated molecules. (**A**) Aromatic rings and (**B**) aliphatic rings.

We examined the generated molecule structures with the highest Tanimoto similarity in six different data distributions generated by the BD-CycleGAN model and Mol-CycleGAN model. It can be observed from Figure 8 that the similarity between X and G(X) of the BD-CycleGAN model and the MolCycleGAN model is generally higher than the similarity between Y and F(Y). The similarity between X and G(X) of the BD-CycleGAN model is higher in the aromatic ring adjustment, while the similarity between Y and F(Y) of the BD-CycleGAN model is higher in the aliphatic ring adjustment. The model may fit in some specific tasks, indicating that a possible modification of BD-CycleGAN is to tune two generators in future work. As shown in Figures S4 and S5, HBA_Continuous obtained the highest similarity score, and the similarity was significantly improved.

**Figure 9:**
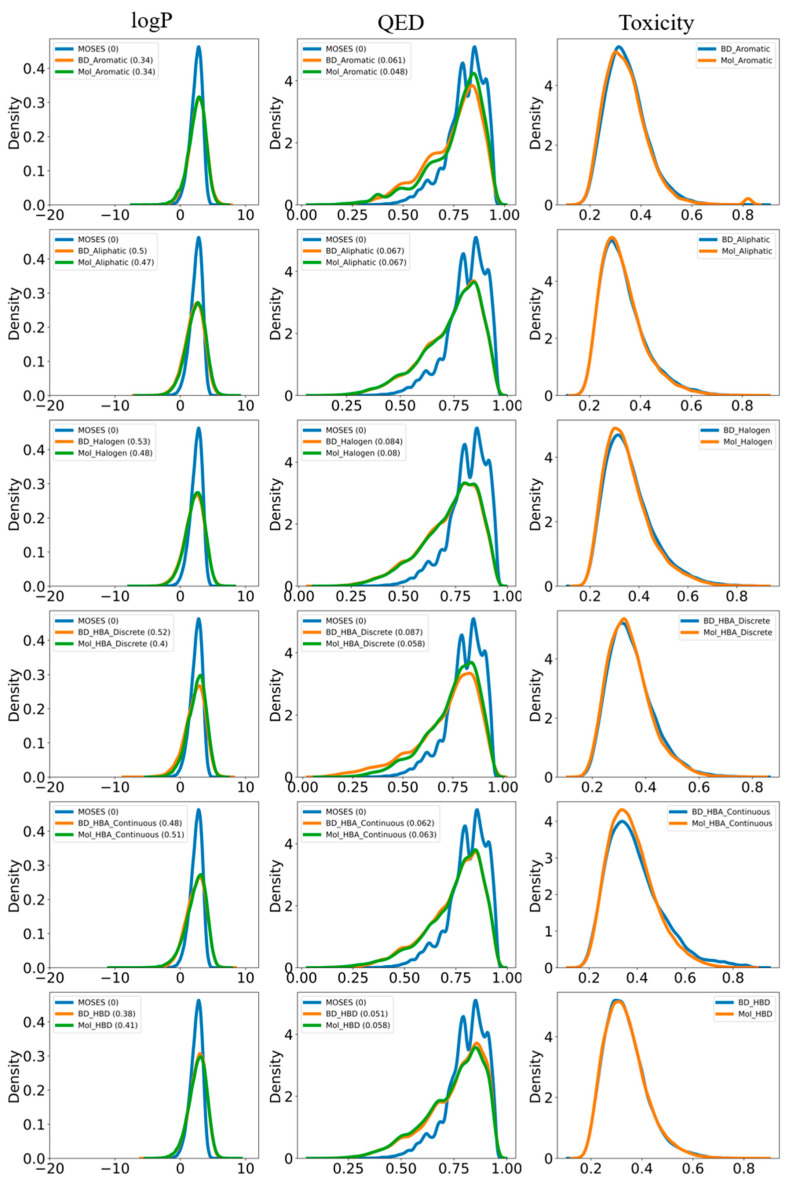
Property distribution of the generated molecules for Mol-CycleGAN and BD-CycleGAN. For clarity, “BD” refers to BD-CycleGAN and “Mol” refers to Mol-CycleGAN. The drug-like properties (logP, QED, and toxicity) are displayed for comparison.

The molecules generated by the BD-CycleGAN model and Mol-CycleGAN model were evaluated according to their structural properties by using the four molecular property indicators: logP, SA, QED, and weight as suggested by MOSES (Figure 9, Figures S7 and S8). The logP metric shows similar distribution for both BD-CycleGAN and Mol-CycleGAN. SA reveals the difficulty of drug synthesis, and it is evident from Figure 9 that aliphatic rings, halogen, and discrete distributed HBA are relatively difficult to synthesize, with halogen being the most difficult. The remaining groups of compounds were less difficult to synthesize. BD-CycleGAN can reduce the synthesizability difficulty for aromatic rings, HBA_continuous, and HBD. From the QED index, it can be seen that the scores of molecules generated by the BD-CycleGAN model are generally higher, which reflects the increase in structural diversity and novelty of the generated molecules, meaning that the generated molecules are closer to drug samples and have higher drug potential and drug feasibility. From the weight index, it can be judged whether the molecules generated by the models are biased towards lighter molecules or heavier molecules. The distribution of weight does not show any obvious difference, except HBA_contiunous, suggesting that Mol-CycleGAN would tend to generate lighter molecules.

In drug design, many factors need to be optimized, such as toxicity. Therefore, we applied eToxPred [32] to compute the toxicity of the generated molecules. Both the BD-CycleGAN and Mol-CycleGAN models generated molecules with comparable levels of toxicity (Figure 9). However, to enhance the toxicity for drug development purposes, it will be necessary to reinforce the generative model by incorporating toxicity as a loss indicator. Future work needs to be conducted to facilitate the improvement of pharmacology properties.

### 2.5. Applications in Active and Decoy Generation

**Figure 10:**
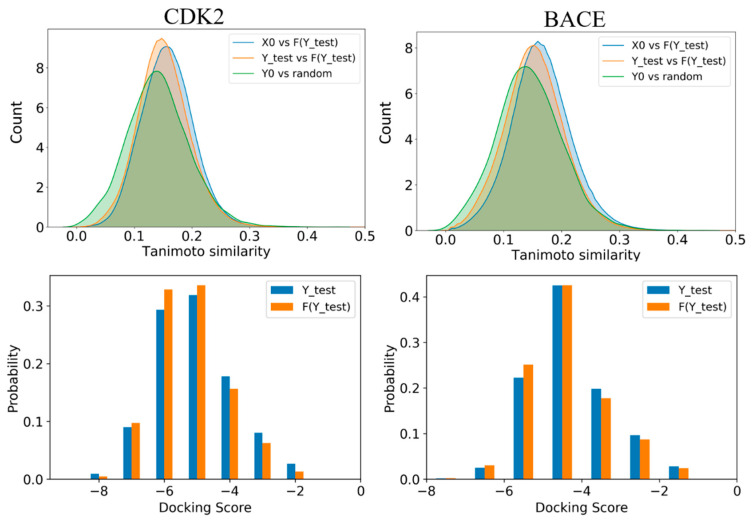
Molecular Tanimoto similarity and distribution of docking scoring for the original and generated molecules in the CDK2 and BACE datasets.

The advantage of BD-CycleGAN is that it inherits the advantage of CycleGAN, which can translate the source domain to the target domain. Therefore, one application of BD-CycleGAN is to perform structural transformations between two datasets; for example, transforming decoys to actives. We assess the model’s generality by applying it to a molecule dataset of cyclin-dependent kinase 2 (CDK2) and the beta-site amyloid precursor protein cleaving enzyme (BACE). The BD-CycleGAN takes the active and decoy compounds as inputs and generates property-matched molecules. As shown in Figure 10, the similarity distribution between the generated molecules of the F(Y_test) and the X molecules surpasses the similarity observed between Y_test molecules and randomly generated molecules (Y_test vs. random). The results demonstrate the applicability of the model, as it is capable of generating molecules with the desired properties and that are structurally similar to the active molecules. The success of such a transformation would depend on the quality and size of the input datasets, and the specific training parameters used for the model. In future, it may be possible to use BD-CycleGAN to generate structurally diverse molecules that have the potential to be active against a given target by carefully training and optimizing the model.

Our proposed model can be used in drug discovery. In this context, the input will be the known inhibitors and the random selected molecules. In the cycle generation, we anticipate that the random molecules, which may not have exhibited inhibitory properties initially, can undergo structural transformations to acquire the desired characteristics of potential inhibitors.

To illustrate the practical application of our approach, we performed molecular docking analyses on both the original molecules and the molecules generated by our model in the target of CDK2 and BACE. The original of molecules for CDK2 and BACE are from the datasets of DUD-E [33] and Enamine [34]. The results, as depicted in Figure 10, reveal a notable trend that the generated molecules (F(Y_test)) exhibit consistently higher docking scores, indicating a stronger binding affinity compared to the original molecules (Y_test). These findings suggest that our generated molecules obtain the pharmacological properties of potential inhibitors that possess favorable interactions with the target molecule of interest.

## 3. Discussion

We propose a molecular generative model called BD-CycleGAN, which embeds BiLSTM and residue connection in the generator and an attention mechanism in the discriminator. By introducing BiLSTM into the generator, the model deals better with the bidirectional dependencies of the sequence data while taking forward and backward contextual information into account, thereby improving the accuracy and consistency of the generated molecules. In addition, by connecting the output of the BiLSTM layer with the original input through the residual connection layer, we can include the original input information in the context features extracted by BiLSTM, preserving the local details and global semantics of the original data. Combining information from the forward and backward directions would enhance the generative ability of the generator. In the discriminator, we embedded an attention layer into the neighboring dense layer. The architecture of discriminator can enhance the feature extraction ability, focus on the key information, and improve the discrimination ability of the discriminator to distinguish between the generated samples and the real samples. Through the attention mechanism, we can determine the importance of each position more precisely and thus better judge the difference between the generated sample and the real one. In summary, the BD-CycleGAN model enables the generator and discriminator to work together more effectively, which improves the quality and accuracy of molecular generation.

Our proposed model can increase the success rate due to the inclusion of BiLSTM and an attention layer. The inclusion of BiLSTM enables the capture of bi-directional dependencies and improves the model’s ability to generate diverse and accurate molecular structures. The attention layer enhances the discriminator’s capability to focus on important patterns in the generated molecules, thereby improving the discrimination process. These architectural enhancements contribute to an increased success rate in generating molecules with desired structural groups. However, it is important to acknowledge the limitations of our proposed model. One notable limitation is its inability to directly optimize pharmacophore properties, such as logP (lipophilicity) and toxicity. In drug discovery, it is crucial to consider not only the structural features of molecules but also their pharmacokinetic and toxicological properties. Incorporating constraints related to properties like logP and toxicity into the molecule generation process is essential to ensuring the viability and safety of potential drug candidates. Therefore, further research is necessary to develop a framework that can generate molecules satisfying both structural requirements and specific property constraints.

## 4. Materials and Methods

### 4.1. BiLSTM for Processing of Bidirectional Molecular Representation

BiLSTM is a bidirectional long short-term memory (LSTM) neural network [35] that can be considered as two LSTMs: a forward LSTM layer and a reverse LSTM layer [36]. In Figure S5, the forward LSTM layer processes the sequence in a forward direction, while the reverse LSTM layer processes the sequence in a backward direction. In the forward LSTM layer, each time step’s input consists of the current input and the hidden state from the previous time step. Conversely, in the reverse LSTM layer, the input from each time step comprises the current input and the hidden state from the subsequent time step. Consequently, the hidden states of the forward and reverse LSTMs are concatenated, yielding a comprehensive representation that serves as the output of the BiLSTM. This combined representation incorporates both forward and backward contextual information, enabling the model to effectively capture long-term dependencies within the sequence.

In molecular generation tasks, the utilization of BiLSTM enables better capturing of information within molecules. More information should facilitate the generation of more accurate and rational molecular sequences. By leveraging contextual information, BiLSTM enhances the prediction of subsequent characters or atoms, ensuring the resulting molecules exhibit sound syntactic and chemical regularity. Its implementation aids in effectively handling challenges such as the vanishing gradient problem and enables a more robust capture of long-term dependencies within the sequence.

### 4.2. Attention Mechanism for Focusing Molecular Information

The attention mechanism is a widely employed technique in deep learning to enhance model performance by dynamically assigning weights to different segments of input data [29,30,31,35,36,37]. In the context of chemical molecular generation tasks, the input strings often tend to be lengthy and challenging to handle. Using attention mechanism can address the bottleneck problem to utilize the most relevant information of the input [38]. In the BD-Cyc1eGAN model, the attention mechanism is incorporated into the discriminator, denoted as D, to facilitate the identification of key features within the input string. By reducing the emphasis on unnecessary information, the attention mechanism contributes to improved accuracy in classification and identification.

### 4.3. Residual Connection for Keeping Molecular Information

Residual connection refers to the introduction of bridge layer connections in the network, which directly transmit the original input to subsequent network layers, thereby enabling faster dissemination and retention of information. Residual connection can effectively preserve the important features and structural information of the original molecule and provide reference and assistance during the generation process. At the same time, residual connections can help alleviate the problem of vanishing or exploding gradients and improve the stability and convergence of model training.

### 4.4. CycleGAN for Molecular Generation in a Cycle Way

The fundamental concept of CycleGAN is to concurrently train two generators and two discriminators. Specifically, one generator is responsible for converting data from one domain to another, while the other generator facilitates the reverse conversion. The purpose of the two discriminators is to assess the authenticity of the generated data. By leveraging the structural characteristics offered by CycleGAN, it becomes possible to achieve precise feature transformations within molecules. Consequently, this capability holds promising potential for facilitating alterations in the properties and characteristics of molecules. As a result, CycleGAN exhibits promising applications in the realms of drug discovery and molecular design.

### 4.5. Model Selection

In molecular generation, the utilization of deep neural networks [39] can enhance the model’s ability to capture information from input sequences, enabling the learning of complex molecular structures and reaction mechanisms. By incorporating BiLSTM, the model can simultaneously consider the information both preceding and following the input sequence, thereby further improving its expressive and predictive capabilities [40]. The integration of attention mechanisms assists the model in focusing on critical segments of the input sequence, leading to more accurate predictions of molecular structures and properties [41]. The combination of various models and methodologies has shown the potential in improving the similarity and success rate of molecular generation. It is important to note that these methods do not guarantee success in all scenarios. In specific applications, careful selection and adjustment of models and techniques are necessary, taking into account the characteristics of the data and tasks at hand. Ablation experiments should be conducted accordingly to achieve the desired outcomes.

Based on a comprehensive analysis of the advantages and limitations of deep neural networks, BiLSTM, attention mechanisms, and LSTM in molecular generation, four experimental groups of models were examined in this study to select the optimal molecule generation models. Figure S1 [see Supplementary Materials] illustrates the algorithm framework for these four models, which share the same discriminator but employ different generators. In the BiLSTM_Attention model, BiLSTM and the attention layer are integrated into the generator. The BiLSTM layer precedes the attention layer, allowing the data to pass through BiLSTM before the attention mechanism assigns weights. Conversely, in the Attention_BiLSTM model, the attention layer is positioned before the BiLSTM layer, assigning weights before the data enter the BiLSTM. The LSTM_Res model incorporates an LSTM module within the generator, where the output of the LSTM layer connects to the embedding layer after passing through the dense layer. Lastly, the BiLSTM_Res model introduces a BiLSTM_block module, where the output of the BiLSTM layer is connected to the embedding layer after traversing the dense layer.

The previous model utilized connected residual layers as the generator and dense layers as the discriminator. After a systematic evaluation of the attention layer, LSTM, and BiLSTM, we introduced an additional BiLSTM layer alongside the residual connection in the generator, aiming to leverage the benefits of bidirectional LSTM for improved performance. We incorporated an attention layer between the dense layers in the discriminator. The addition of the attention layer in the discriminator helps to effectively capture important patterns in the generated molecules.

### 4.6. Workflow

Generation models typically use simplified molecular input line entry system (SMILES) [42,43] characters and molecular diagrams [44] to generate target molecules. However, SMILES would suffer from the problem of generating invalided molecules. Therefore, we select JT-VAE as the encoding method to ensure the generated molecules remain valid. Before model training, the SMILES are mapped to JT-VAE space.

We maintained the symmetric structure of CycleGAN in our approach. As illustrated in Figure 2, we constructed and presented two identical generators and two discriminators in a symmetrical fashion. The generator component incorporates BiLSTM as a crucial element. Initially, the potential vector obtained from the potential spatial sampling of JTVAE serves as the input for BD-CycleGAN model. The bidirectional loop architecture of BiLSTM aids in capturing contextual information about the input vectors and generating more comprehensive feature representations. Subsequently, the output of BiLSTM is dimensionally transformed through a dense layer and then fused with the original input vectors to incorporate the information from both the potential vectors and the original inputs, thereby enhancing the generator’s capabilities.

The discriminator utilizes a stacked structure consisting of a dense layer and an attention mechanism layer, which is stacked a total of three times. In each stacking process, input vectors undergo feature extraction and dimension transformation through the dense layer, resulting in a set of low-dimensional feature representations. Subsequently, the attention mechanism calculates the weights for each position within this feature set, and the weighted sum of these attention weights yields the final representation of the feature set. This stacking operation is repeated three times, progressively extracting and fusing features, thereby enhancing the discriminator’s sensitivity to the distinctions between the generated and the real molecules. As a result, discrimination accuracy and effectiveness are improved. The discriminator designed with this structure effectively discerns the generated molecules from the real ones, thereby enhancing the discriminative capability of the model.

### 4.7. Evaluation Metrics

We evaluated the model by using MOSES [45] metrics to measure the molecular structure, chemical properties, and overall similarity of the resulting molecular assemblages. The nearest neighbor similarity, fragment similarity, scaffold similarity, valley similarity, and Fréchet ChemNet Distance were calculated to evaluate the performance of the model. These indicators can help us understand how similar the generated molecule is to the target molecule, how well the chemical properties match, and how consistent the overall structure is. The evaluation indexes are provided in the Supplementary Materials. Through these evaluation indexes, we can more comprehensively evaluate the performance of the model in molecular generation tasks.

### 4.8. Data Set

The commonly used ZINC-250K is selected as our dataset. We select the molecules based on the functional groups. Pharmacodynamic functional groups refer to the structural units that have certain chemical properties and can affect the biological activity of molecules. The presence or absence of pharmacodynamic functional groups affects the biological activity of a molecule. Therefore, the identification and analysis of pharmacodynamic functional groups are very important in the process of drug discovery. The classification of pharmacodynamic functional groups can be carried out according to their presence, location, and number in the molecule. The classification method based on pharmacodynamic functional groups classifies molecules according to their structural characteristics, and further studies the relationship between structural characteristics and biological activities. This classification method is highly interpretable and structurally specific and can help to better understand the relationship between molecular structure and biological activity.

The pharmacodynamic category is defined by the presence or absence of these functional groups. The ZINC-250K data set was divided into training sets, validation sets, and test sets, and the number of different functional groups was selected as the basis for division. As shown in Figure S3, the ZINC-250K data set was divided into six data sets based on the number of five functional groups: aromatic ring, aliphatic ring, halogen, hydrogen bond donor (HBD), and hydrogen bond acceptor (HBA).

Aromatic Rings: Molecules with only two aromatic rings in the X dataset, while molecules with one or three aromatic rings belong in the Y dataset.

Aliphatic Rings: Molecules with only one aliphatic ring in the X dataset and two or three aliphatic rings in the Y dataset.

Halogen: Molecules in the X dataset contain no halogens (F, Cl, Br, I and CN), while molecules in the Y dataset have one, two, three, four, or five halogens.

HBD: The molecules in the X dataset contained only one hydrogen bond donor, while the molecules in the Y dataset had zero, two, three, four, or five hydrogen bond donors.

To further demonstrate the generation ability, we conduct a task by using the discrete number and the continuous number of HBA.

HBA_Discrete: The molecules in the X data set contain only five hydrogen bond acceptors, while molecules in the Y data set have zero, two, three, or four hydrogen bond acceptors.

HBA_Continuous: Molecules in the X data set have zero, one, two, three, four, or five hydrogen bond receptors, while molecules in the Y data set have 6, 7, 8, 9, or 10 hydrogen bond receptors.

Aromatic rings and aliphatic rings are used to tune the hydrophobic property in the lead optimization. HBA and HBD represent the functional group of a molecule where protons can form hydrogen bonds with other molecules, thus they play key roles in the recognition, binding, and permeation of molecules through the cell membrane. The efficiency and biological feasibility of generating molecules can be improved by changing functional groups.

Our proposed method is limited to altering the number of functional groups rather than changing the types of functional groups. This limitation is worth noting. Specifically, our approach utilizes neural network-based models to modify the quantity of functional groups in molecules. For instance, we can adjust the number of aromatic rings, aliphatic rings, halogens, hydrogen bond donors, and hydrogen bond acceptors.

### 4.9. Applications in Active and Decoy Generation

The proposed model can achieve cyclic molecule generation. Therefore, we assessed its application in generating active molecules from decoy compounds. We chose two popular targets for our test, these being cyclin-dependent kinase 2 (CDK2) and beta-site amyloid precursor protein cleaving enzyme (BACE). The CDK2 dataset consists of 474 actives and 27850 decoys. BACE consists of 7172 potential inhibitors from the Enamine BACE-targeted library and 7172 random selected molecules from ZINC-250K. We converted the active and decoy compounds into the JT-VAE input and generated property-matched molecules. The active molecules were labeled as X and the decoys were labeled as Y.

## 5. Conclusions

We proposed the BD-CycleGAN model to improve the generative ability of de novo molecule generation. The performance of our model is evaluated by generating six sets of molecules with different structural features. The results show that incorporating BiLSTM and residual connection into the generator can improve the success rate by effectively handling the bidirectional information in the sequence data. The revisions made to the generator and discriminator as part of the cycle-type GAN can further enhance the bidirectional generative ability. The experimental findings highlight two key advantages of the BD-CycleGAN model. Firstly, it exhibits improved diversity in the generated molecules, allowing for a broader exploration of chemical space. Additionally, the model achieves increased similarity between the source molecules and the generated molecules, which is required for the lead optimization process in drug design. Overall, our BD-CycleGAN model achieves better performance in terms of molecular generation, which provides a promising tool for the molecular design and discovery.

## Figures and Tables

**Table 1:** Assessment of Structural Modifications in Generated Molecules.

Model		X—>G(X)			Y—>F(Y)		
Data	Model	Success Rate	Diversity	Non-Identity	Success Rate	Diversity	Non-Identity
Aromatic rings	Mol-CycleGAN	0.231	0.981	0.762	0.102	0.997	0.582
	BD-CycleGAN	0.257	0.995	0.804	0.117	0.997	0.680
Aliphatic rings	Mol-CycleGAN	0.183	0.996	0.713	0.154	0.994	0.769
	BD-CycleGAN	0.222	0.996	0.814	0.196	0.996	0.861
Halogen	Mol-CycleGAN	0.032	0.997	0.417	0.145	0.993	0.717
	BD-CycleGAN	0.121	0.994	0.714	0.257	0.991	0.793
HBD	Mol-CycleGAN	0.226	0.994	0.718	0.147	0.991	0.822
	BD-CycleGAN	0.193	0.996	0.782	0.178	0.996	0.923
HBA_Discrete	Mol-CycleGAN	0.154	0.986	0.389	0.030	0.999	0.328
	BD-CycleGAN	0.376	0.995	0.782	0.078	0.996	0.662
HBA_Continuous	Mol-CycleGAN	0.106	0.995	0.518	0.102	0.994	0.475
	BD-CycleGAN	0.085	0.974	0.662	0.142	0.966	0.662

**Table 2:** The evaluation of generated molecules in terms of success rate and the validity of molecules that meet chemical rules and chemical diversity.

Structure	Model	X—>G(X)					Y—>F(Y)				
		Filters	Valid	IntDiv	IntDiv2	Novelty	Filters	Valid	IntDiv	IntDiv2	Novelty
Aromatic	Mol-Cycle	0.621	0.989	0.865	0.859	0.954	0.598	0.998	0.869	0.863	0.947
	BD-Cycle	0.639	0.995	0.866	0.860	0.961	0.599	0.998	0.868	0.863	0.957
Aliphatic	Mol-Cycle	0.576	0.998	0.862	0.857	0.960	0.518	0.994	0.865	0.859	0.974
	BD-Cycle	0.585	0.997	0.863	0.857	0.970	0.519	0.996	0.867	0.861	0.980
Halogen	Mol-Cycle	0.587	0.998	0.869	0.863	0.927	0.618	0.994	0.865	0.859	0.963
	BD-Cycle	0.576	0.998	0.873	0.867	0.960	0.534	0.994	0.870	0.864	0.975
HBD	Mol-Cycle	0.691	0.995	0.865	0.859	0.950	0.549	0.993	0.871	0.866	0.975
	BD-Cycle	0.699	0.997	0.863	0.857	0.956	0.554	0.997	0.874	0.868	0.986
HBA_Discete	Mol-Cycle	0.636	0.988	0.865	0.859	0.905	0.519	0.999	0.882	0.874	0.943
	BD-Cycle	0.611	0.996	0.868	0.862	0.956	0.454	0.999	0.887	0.879	0.965
HBA_Continuous	Mol-Cycle	0.533	0.997	0.880	0.873	0.939	0.647	0.997	0.863	0.857	0.940
	BD-Cycle	0.589	0.998	0.882	0.874	0.953	0.649	0.997	0.866	0.858	0.952

Mol-Cycle is the abbreviation for Mol-CycleGAN, BD-Cycle is the abbreviation for BD-CycleGAN.

**Table 3:** The evaluation of generated molecules in terms of structural similarity and novel molecular fragment similarity in the process from X to G(X).

X—>F(X)		FCD		SNN		Scaff		Frag	
Data	Model	Test	TestSF	Test	TestSF	Test	TestSF	Test	TestSF
Aromatic Rings	Mol-CycleGAN	0.627	4.487	0.609	0.467	0.901	0.143	0.998	0.909
	BD-CycleGAN	0.831	4.460	0.578	0.466	0.887	0.149	0.997	0.989
Aliphatic Rings	Mol-CycleGAN	0.278	5.971	0.669	0.466	0.922	0.125	0.999	0.990
	BD-CycleGAN	0.464	6.275	0.603	0.456	0.895	0.098	0.999	0.990
Halogen	Mol-CycleGAN	0.082	5.397	0.825	0.480	0.942	0.200	0.999	0.987
	BD-CycleGAN	0.591	5.700	0.640	0.448	0.847	0.170	0.997	0.986
HBD	Mol-CycleGAN	0.419	4.08	0.653	0.476	0.907	0.138	0.998	0.993
	BD-CycleGAN	0.368	4.126	0.637	0.476	0.904	0.143	0.999	0.993
HBA_Discrete	Mol-CycleGAN	0.160	4.016	0.882	0.498	0.901	0.143	0.999	0.993
	BD-CycleGAN	0.864	4.833	0.596	0.461	0.717	0.145	0.998	0.991
HBA_Continuous	Mol-CycleGAN	0.212	6.288	0.734	0.450	0.957	0.196	0.997	0.968
	BD-CycleGAN	0.562	5.944	0.692	0.450	0.883	0.191	0.997	0.982

**Table 4:** The evaluation of generated molecules in terms of structural similarity and novel molecular fragment similarity in the process from Y to F(Y).

Y—>F(Y)		FCD		SNN		Scaff		Frag	
Data	Model	Test	TestSF	Test	Test	Test	TestSF	Test	TestSF
Aromatic Rings	Mol-CycleGAN	0.135	4.741	0.727	0.469	0.908	0.104	0.999	0.990
	BD-CycleGAN	0.175	4.915	0.675	0.465	0.887	0.101	0.999	0.991
Aliphatic Rings	Mol-CycleGAN	0.494	10.810	0.607	0.438	0.433	0.011	0.998	0.971
	BD-CycleGAN	0.633	10.403	0.538	0.428	0.371	0.018	0.997	0.974
Halogen	Mol-CycleGAN	0.558	5.778	0.638	0.462	0.841	0.135	0.998	0.984
	BD-CycleGAN	2.153	7.560	0.551	0.433	0.605	0.148	0.982	0.975
HBD	Mol-CycleGAN	0.358	5.553	0.594	0.447	0.904	0.204	0.997	0.988
	BD-CycleGAN	0.758	6.229	0.513	0.429	0.840	0.172	0.994	0.986
HBA_Discrete	Mol-CycleGAN	0.034	8.755	0.862	0.452	0.982	0.179	0.999	0.941
	BD-CycleGAN	0.470	10.012	0.674	0.422	0.927	0.182	0.977	0.880
HBA_Continuous	Mol-CycleGAN	0.272	4.975	0.755	0.483	0.824	0.106	0.999	0.994
	BD-CycleGAN	0.543	4.721	0.673	0.476	0.666	0.109	0.999	0.993

## Data Availability

The dataset supporting the conclusions of this article is available in the ZINK250K repository, hyperlink to dataset(s): https://www.kaggle.com/datasets/basu369victor/zinc250k (accessed on 6 December 2021. The scripts of this article is available in the GitHub repository, in https://github.com/AIMedDrug/BD-CycleGAN (accessed on 15 September 2023).

## Supplementary Materials

The following supporting information can be downloaded at: https://www.mdpi.com/article/10.3390/molecules29071499/s1, Figure S1: Schematic diagram of the four combined models.; Figures S2 and S3: molecular distribution; Figures S4 and S5: structure of generated molecules; Figure S6: schematic figure of BiLSTM; Figure S7: Synthetic Accessibility (SA) distribution of generated molecules; Figure S8: Molecular weight distribution of generated molecules.
