# Supplementary material for: Developing an Improved Cycle Architecture for AI-Based Generation of New Structures Aimed at Drug Discovery
Source: Molecules. 2024 Mar 27;29(7):1499. doi: 10.3390/molecules29071499 (PMC11013495; doi:10.3390/molecules29071499)
Supplement: Supplementary file 1 [file molecules-29-01499-s001.zip › molecules-2865385-supplementary.pdf]

# Developing An Improved Cycle Architecture for AI-based Generation of New Structures Aimed at Drug Discovery

Chun Zhang#, Liangxu Xie#, Xiaohua Lu, Rongzhi Mao, Lei Xu*, Xiaojun Xu*

Institute of Bioinformatics and Medical Engineering, School of Electrical and Information Engineering, Jiangsu University of Technology, Changzhou 213001, China.

**Supplementary computational details and figures.**

**Evaluation indicators**

**Success rate**: Calculate the success rate of generating target molecules by calculating the number of target molecules in the whole. A higher success rate indicates that the molecules generated by the model are more consistent with the expected target and have higher practicability.

**Diversity**: Diversity indicators reflect whether the generated molecules have diverse structural and chemical properties. Higher diversity means that the resulting molecules have greater structural variation and chemical spatial coverage.

**Non-identity**: Non-repeatability indicators measure the degree of difference between molecules in the generated set of molecules. A higher non-repeatability indicates that the molecules in the resulting set of molecules are more unique and not the duplicated or highly similar molecules.

**Filters**: The Filters metric measures the percentage of generated molecules that pass a

predefined filter condition. These filter conditions can be rules, restrictions, or targets used to screen out molecules that meet specific requirements. A higher value of the filter indicator indicates that the generated molecules meet the expected requirements and have the required characteristics.

**Valid**: The Valid metric measures the percentage of generated molecules that meet chemical rules and constraints. An efficient molecule complies with chemical stability, rules for atomic valence bonds, and other chemicals constrain. A higher Valid index indicates that the molecules generated are more likely to be chemically reasonable and viable molecules.

**IntDiv**: IntDiv metrics evaluate the internal diversity in the generated collection of molecules. A higher IntDiv value indicates that the resulting collection of molecules is more diverse, containing more different structures.

**IntDiv2**: IntDiv2 is an improved version of IntDiv that further considers the properties and properties of molecules. In addition to structural differences, IntDiv2 also focuses on differences in the chemical properties of molecules. A higher IntDiv2 value indicates that the resulting set of molecules is more diverse in both structure and properties.

**Novelty**: The Novelty index measures the novelty in the collection of molecules produced. A higher Novelty value indicates that the generated molecule is more innovative and unique.

**logP**: The logP is a measure of the lipid solubility or hydrophilicity of a molecule. It represents the distribution of molecules between the oil and water phases and is often used to predict drug absorbability and metabolic activity of molecules. A higher logP

value indicates that the molecule is hydrophobic, while a lower logP value indicates that the molecule is more hydrophilic.

Synthetic accessibility **(SA)**: SA is an indicator to evaluate the synthesizability of molecules. The smaller, the better.

**QED** (Quantitative Estimation of Drug-likeness): QED is a measure of the similarity of molecular drugs, which is used to evaluate whether the molecule has drug-like characteristics. A higher QED value indicates that the molecule is more drug-like and has more potential to be a drug candidate.

**Weight**: Molecular weight refers to the total atomic mass of a molecule and is used to measure its size and complexity. Molecular weight plays an important role in the feasibility and performance of drug discovery and chemical synthesis. Lower molecular weights are generally associated with better drug absorption, metabolism, and delivery performance.

**Tanimoto Similarity**: Tanimoto similarity is a common molecular similarity measure used to compare the structural similarity between two molecules. Higher valley similarity indicates that two molecules have greater structural similarity.

**FréchetChemNet Distance (FCD)**: The performance of the model on the overall molecular structure is evaluated by calculating the Fréchet ChemNet distance between the generated molecular set and the target molecular set. When the value is greater than zero, the smaller the FCD value is, the more similar the generated molecule set is to the target molecule set.

Its principle is shown in the following equation:

$$FCD(G,R) = \|\mu_G - \mu_R\|^2 + Tr(\sum G + \sum R - 2(\sum G \sum R)^{1/2}) \quad (1)$$

where $\mu_G$, $\mu_R$ are mean vectors and $\sum G$, $\sum R$ are the total covariance matrix matrices of activations for molecules.

from sets G and R respectively.

**Substructure-non-substructure Similarity** (SNN): The SNN measure is utilized to evaluate the degree of similarity between the generated molecules and the target molecules at the substructural level. It quantifies the substructural similarity between each molecule in the generated molecule set and the target molecule set and then computes the average. A higher SNN value indicates that the generated molecule effectively preserves the crucial substructures present in the target molecule.

Its principle is shown in the following equation:

$$SNN(G,R) = \frac{1}{|G|} \sum_{m_G \in G} \max_{m_R \in R} T(m_G, m_R) \quad (2)$$

$T(m_G, m_R)$ is the generated set *G* molecule $m_G$ and the nearest neighbor molecule $m_R$ in the true dataset *R*.

**Scaffold Similarity (Scaff)**: The scaffold similarity between the generated molecule and the target molecule is evaluated. It calculates the Scaffold similarity between each molecule in the generated set of molecules and the target set of molecules and then averages it. A higher Scaff value indicates that the generated molecule can retain the key skeleton structure of the target molecule.

Its principle is shown in the following equation:

$$Frag(G,R) = \frac{\sum_{s \in S}(c_s(G) \bullet c_s(R))}{\sqrt{\sum_{s \in S} c_s^2(G)} \sqrt{\sum_{s \in S} c_s^2(R)}} \quad (3)$$

The number of times scaffold $s$ appears in the molecules of set A is represented as $c_s(A)$, and the set of fragments that appear in *G* or *R* is represented as *S*, with the metric defined as cosine similarity.

**Fragment Similarity (Frag):** Frag is used to measure the fragment similarity between the generated molecule and the target molecule. It computes the fragment similarity between each molecule in the generated set of molecules and the set of target molecules and then averages it. A higher Frag value indicates that the generated molecule can retain important segments of the target molecule.

Its principle is shown in the following equation:

$$Frag(G,R)=\frac{\sum_{f\in F}(c_f(G)\cdot c_f(R))}{\sqrt{\sum_{f\in F}c_f^2(G)}\sqrt{\sum_{f\in F}c_f^2(R)}} \quad (4)$$

The $c_f(A)$ represents the number of times substructure $f$ appears in the molecules of set A, and the set of fragments that appear in G or R is F, with the metric defined as cosine similarity.

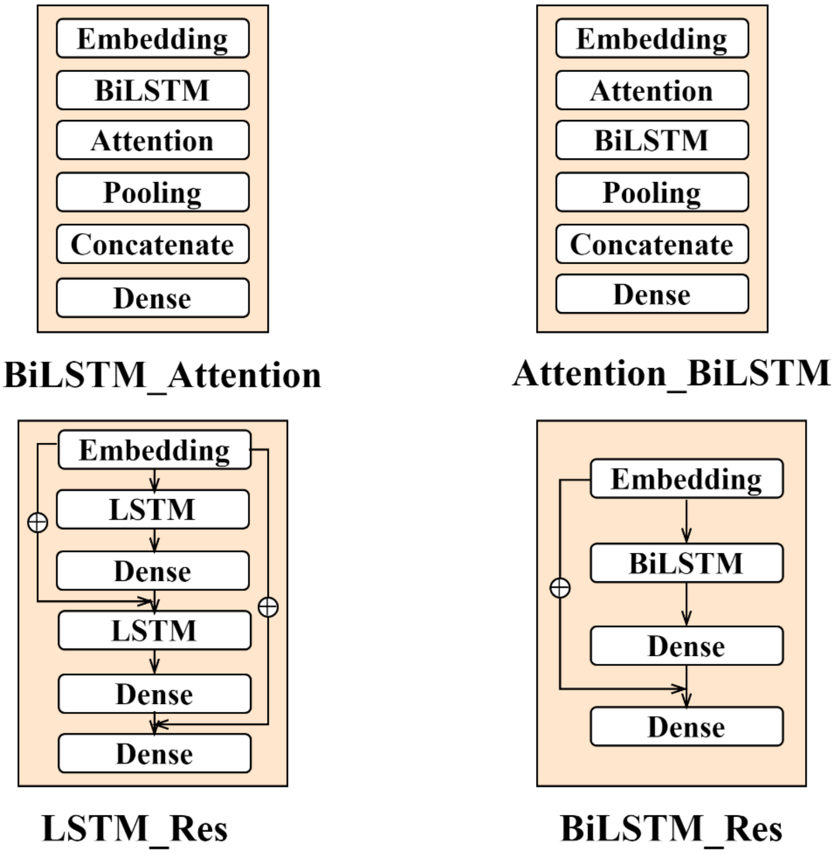


**Figure S1.** Schematic diagram of the four combined models. BiSTM_Attention refers to BiLSTM layer preceding the Attention layer; Attention_BiLSTM refers to Attention layer preceding the BiLSTM layer; LSTM_Res refers to LSTM layer connected by residual connection; BiLSTM_Res refers to BiLSTM layer connected with residual connection.

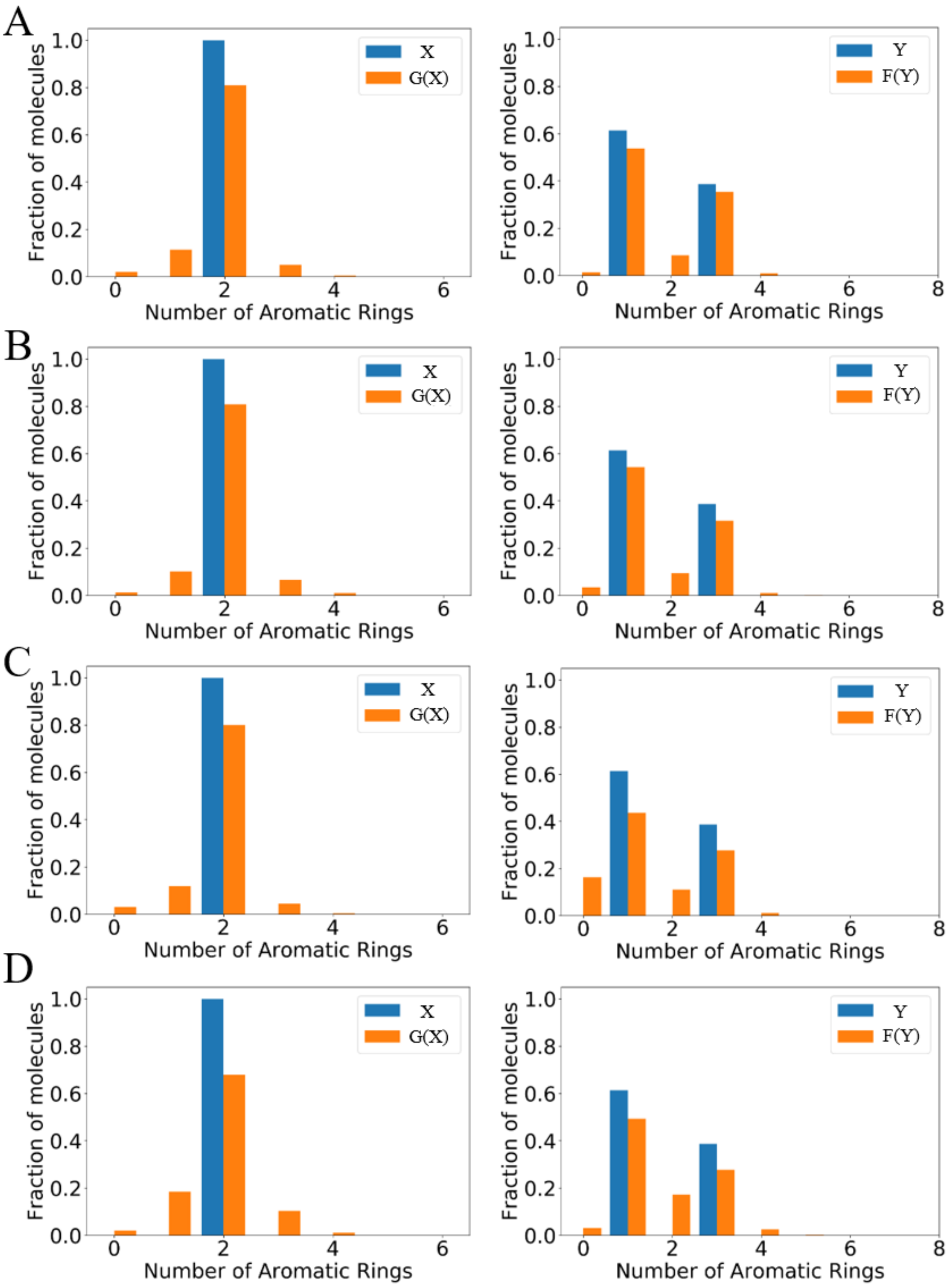


**Figure S2**. Molecular distribution maps generated by four combined models. A is the BiLSTM-Attention combination model, B is the Attention_BiLSTM combination model, and C is the LSTM_Res combination model. D is the BiLSTM_res.

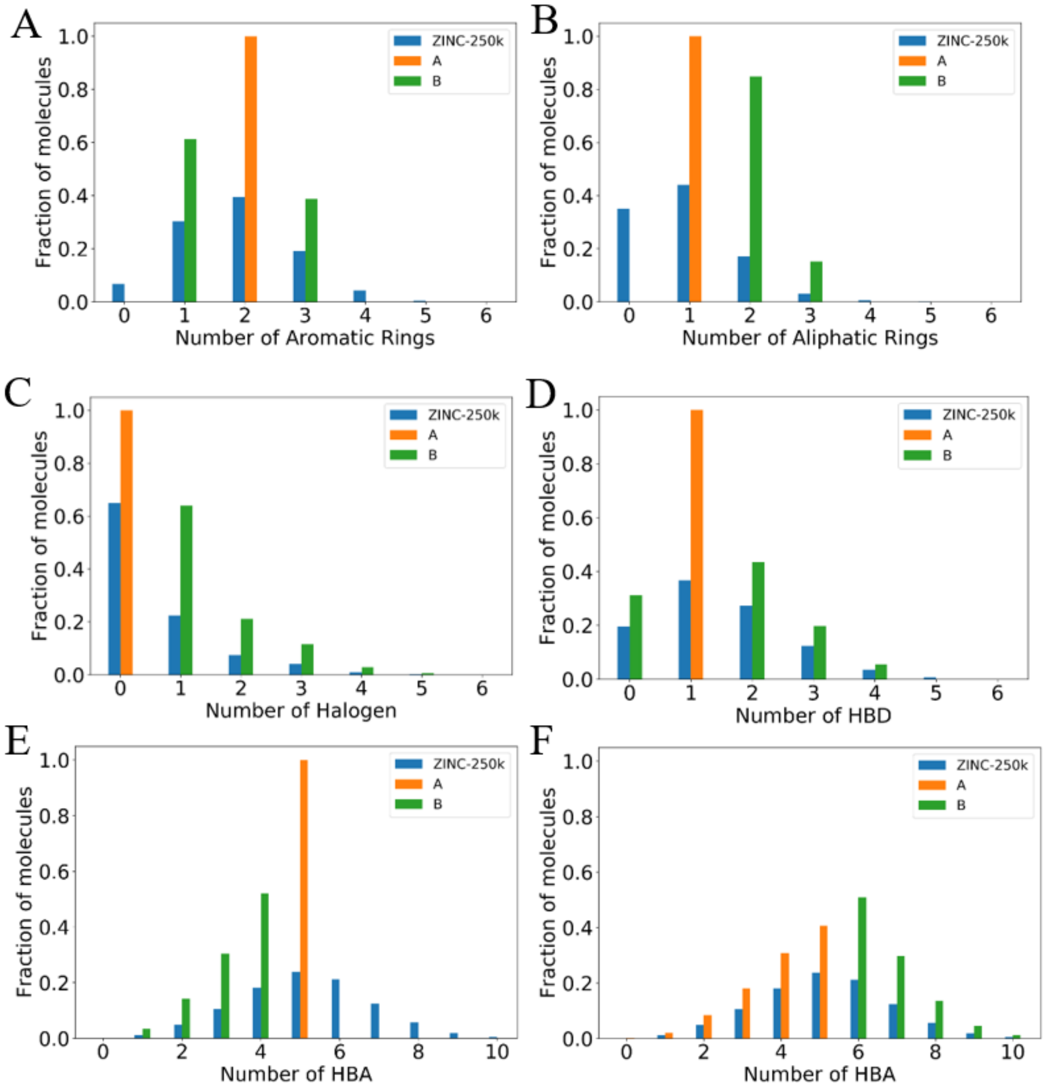


**Figure S3** The distribution of molecules in the X data set and Y data set. Orange represents the molecular distribution of the X dataset, green represents the molecular distribution of the Y dataset, and represents the distribution of each functional group under the ZINC-250K dataset. A is for Aromatic Rings, B is for Aliphatic Rings, C is for Halogen, D is for HBD, E is for HBA_Discrete, and F is for HBA_Continuous.

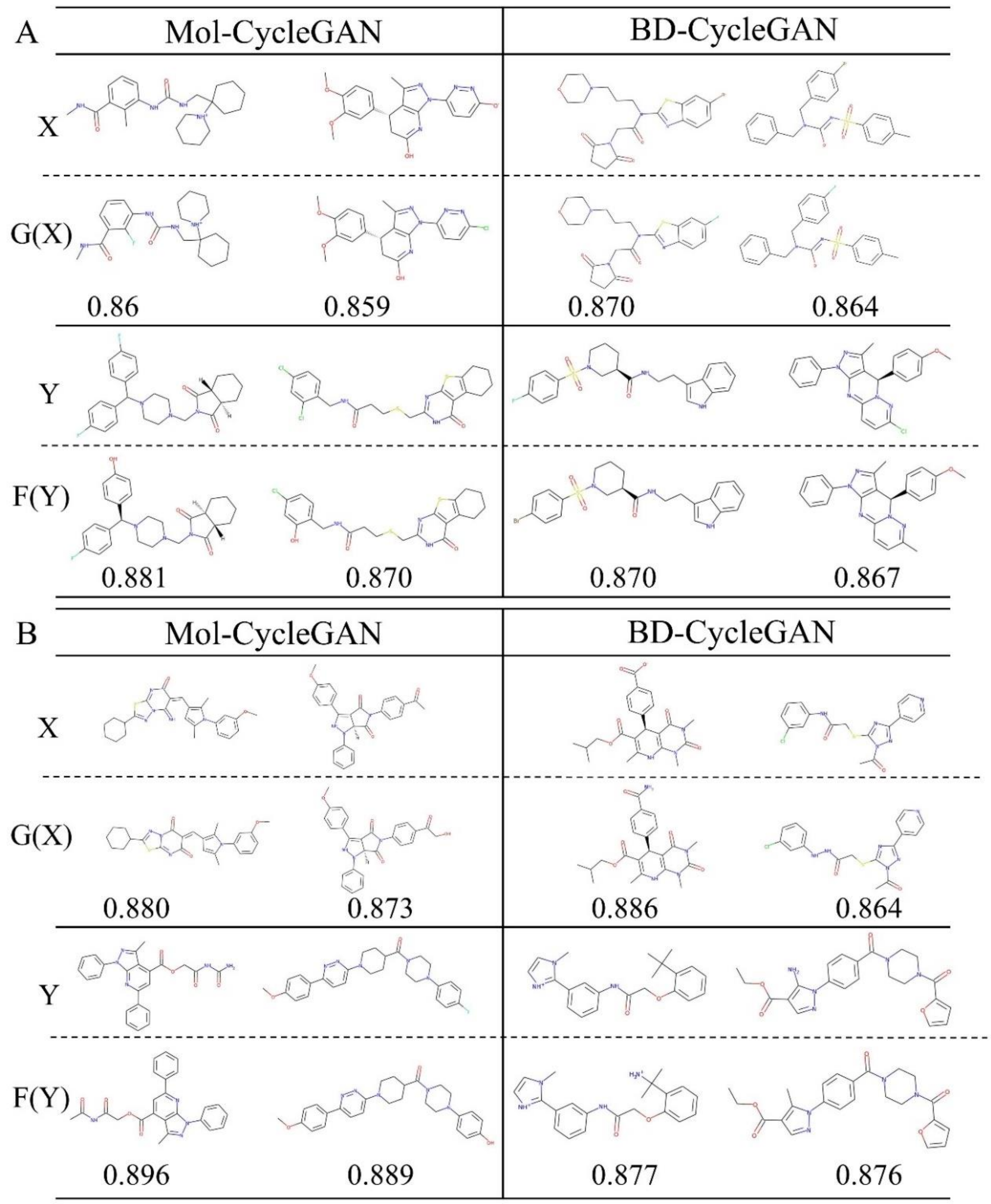


**Figure S4.** Structure diagram of the original and generated molecules. A is Halogen and B is HBD.

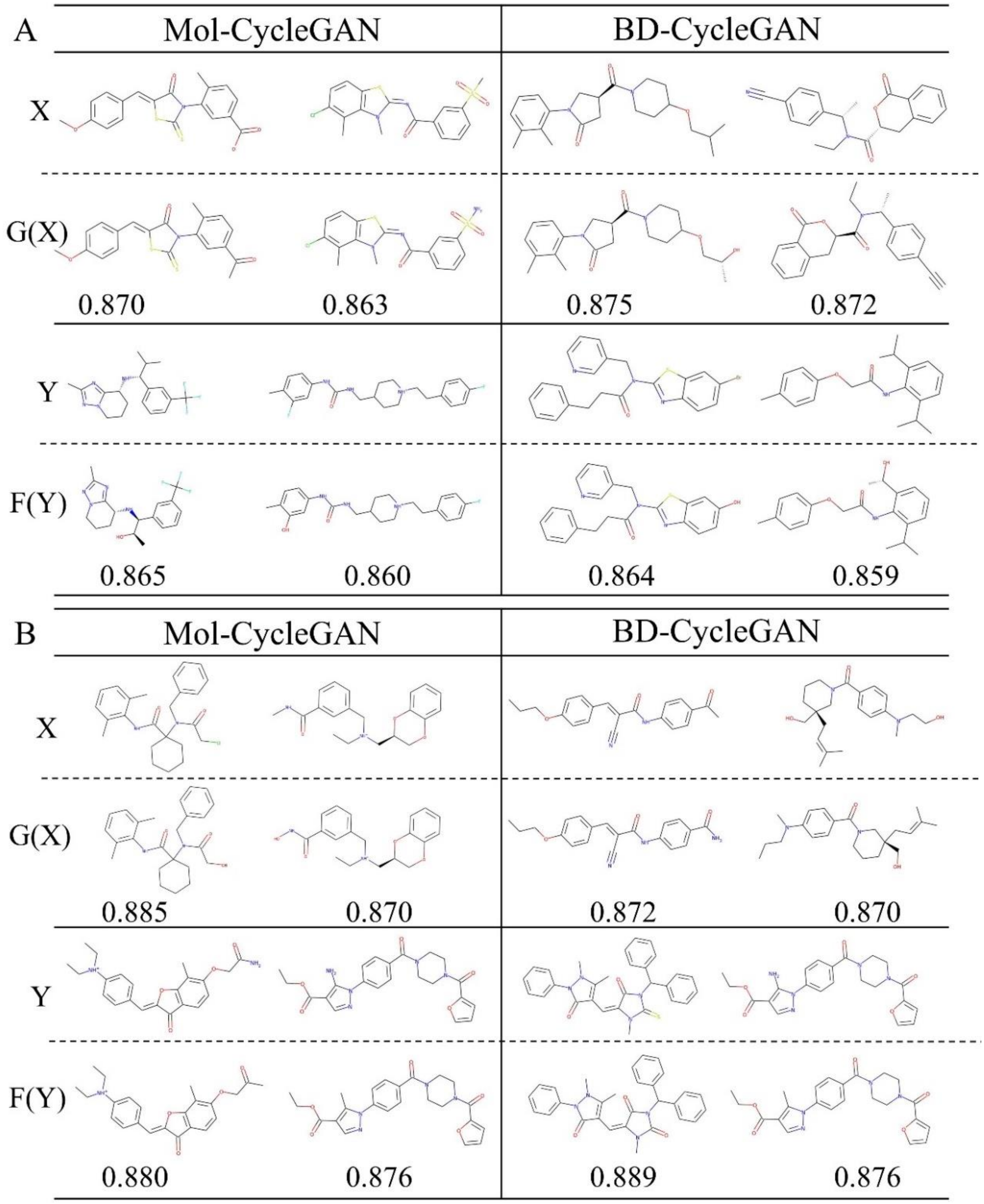


**Figure S5.** Structure diagram of the original and generated molecules. A is HBA_Discrete and B is HBA_Continuous.

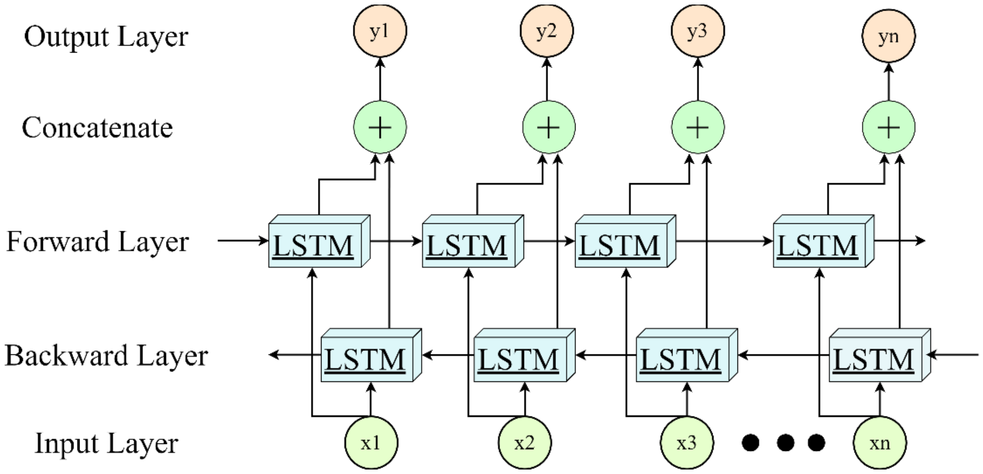


**Figure S6**. Schematic figure of BiLSTM neural network.

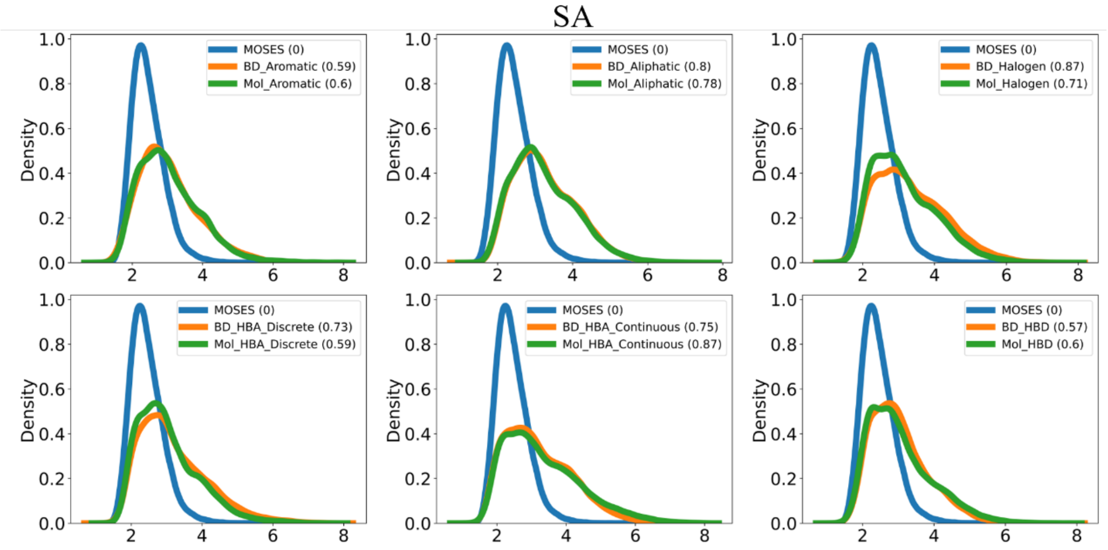


**Figure S7**. Synthetic Accessibility (SA) distribution of generated molecules for Mol-CycleGAN and BD-CycleGAN. For clarity, "BD" refers to BD-CycleGAN and "Mol" refers to Mol-CycleGAN.

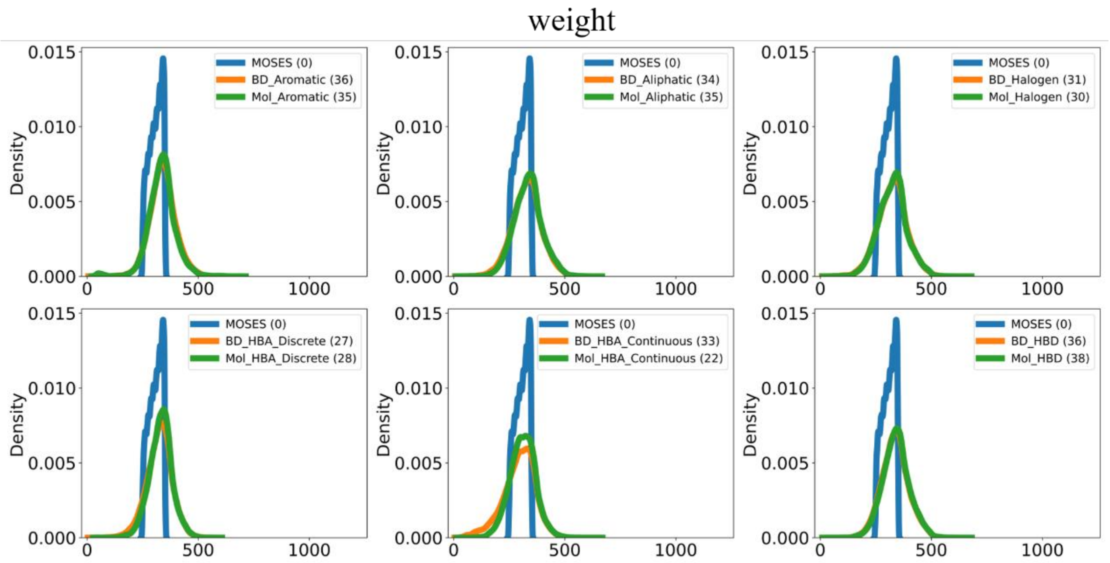


**Figure S8** Molecular weight distribution of generated molecules for Mol-CycleGAN and BD-CycleGAN. For clarity, "BD" refers to BD-CycleGAN and "Mol" refers to Mol-CycleGAN.
